# Supplementary material for: Optogenetically stimulating intact rat corticospinal tract post-stroke restores motor control through regionalized functional circuit formation
Source: Nat Commun. 2017 Oct 30;8:1187. doi: 10.1038/s41467-017-01090-6 (PMC5662731; doi:10.1038/s41467-017-01090-6)
Supplement: Supplementary file 1 — Supplementary information [file 41467_2017_1090_MOESM1_ESM.pdf]

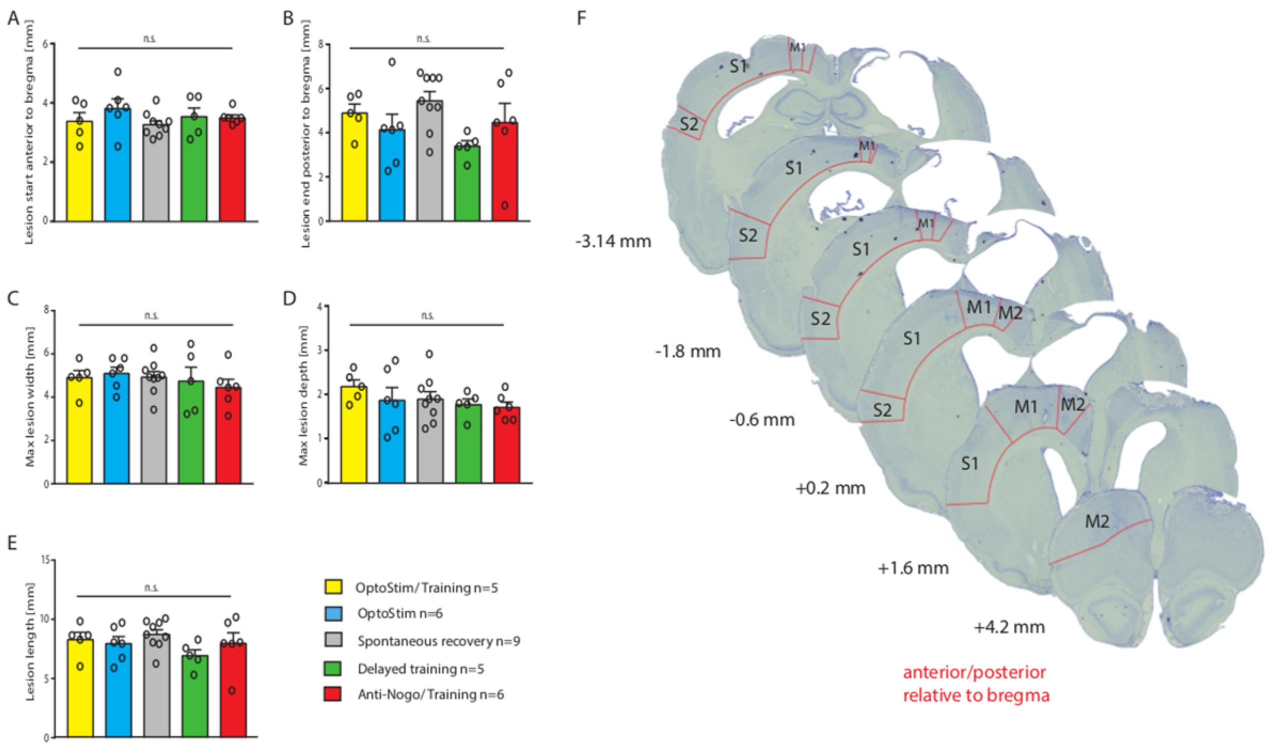


**Supplementary Figure 1.** Detailed stroke lesion analysis revealed no difference concerning the stroke lesion position (A= lesion start anterior to bregma, B= lesion end posterior to bregma), maximal lesion width (C) and depth (D) as well as lesion length (E) among the five different rehabilitation groups (‘OptoStim/Training‘, ‘OptoStim‘, ‘Spontaneous recovery‘,‘Delayed Training‘, ‘Anti-Nogo/Training‘). Data are presented as means ± s.e.m.: statistical evaluation was carried out with one-way ANOVA followed by Bonferroni post hoc (n.s.= not significant). (F) Representative series of coronal Nissl stained brain sections depicting stroke lesion dimension and location relative to bregma. M1= primary motor cortex, M2= premotor cortex, S1= primary sensory cortex, S2= secondary sensory cortex.

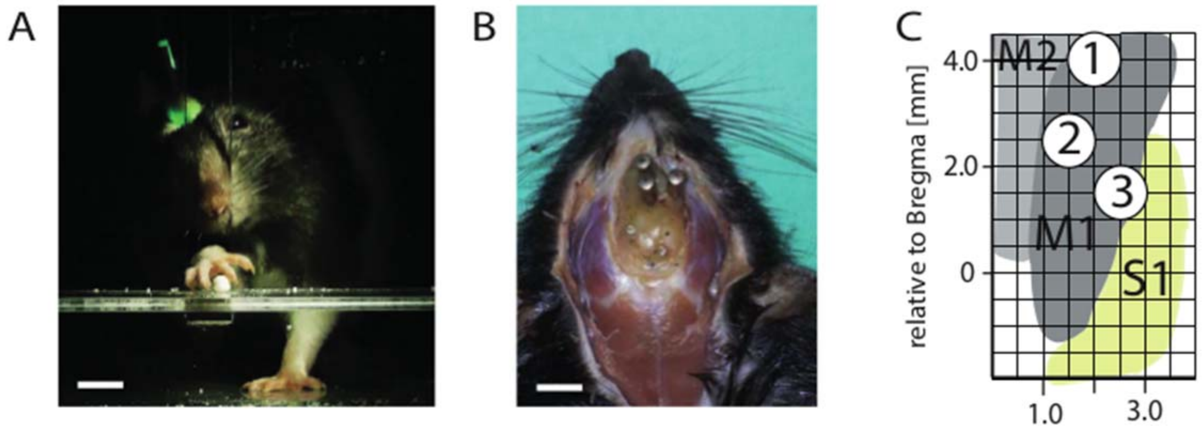


**Supplementary Figure 2.** (A) Image depicting a grasping rat which is attached to light stimulation through the three optic implants over the pre- and primary motor corex (scale bar= 1cm. (B) Representative image showing the optic implants mounted on top of the skull with dental cement and permanently fixed with 3-4 screws to the skull (scale bar= 6 mm). (C) Scheme revealing the positions of the three optical implants (1-3) covering an estimated quarter (24.8%) of the primary motor cortex surface.

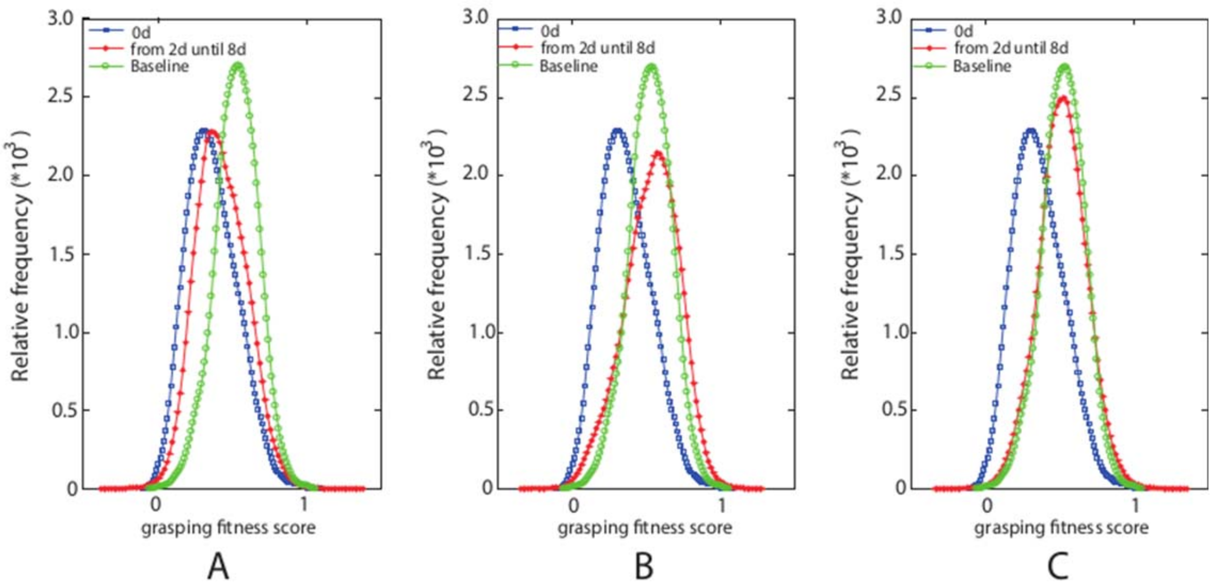


D P-values between 0d and all time ranges

| | 0d |
|---|---|
| From 2d until 8d | 0.0474541785 |
| From 14d until 17d | 2.46E-014 |
| From 18d until 14d | 4.44E-018 |

**Supplementary Figure 3**. Analysis of the grasping behavior during the initial learning of the skilled learning task. For 25 animals frequent recordings documented within a period of 30 days (pre-stroke) are evaluated using our kinematic analysis. We then map each grasp to a score, which indicates grasping quality: Therefore, we train a classifier using a subset of grasps from before the learning has started (0 days, marked as blue squares) and grasps after learning and right before surgery (baseline, marked as red stars), they are mapped to 0 and 1, respectively. Thereafter the classifier is used to assign all the grasps from all dates to a score. The figure shows the relative frequency of grasping fitness scores for the different dates. A score close to 0 implies a high similarity to grasping trials before learning, whereas a score around 1 indicates a high affinity to baseline sequences. It can be seen in (A) that after only a few days of training (after 2 o 8 days) the animals start to improve in the skilled reaching task. From the second week on (B) the rats already reached a high standard grasping behavior, which improves even more during the last few days of training (C). The p-values (K-S-Test) shown in (D) confirm, that the algorithm has correctly identified the changes in grasping during even short phases of learning.

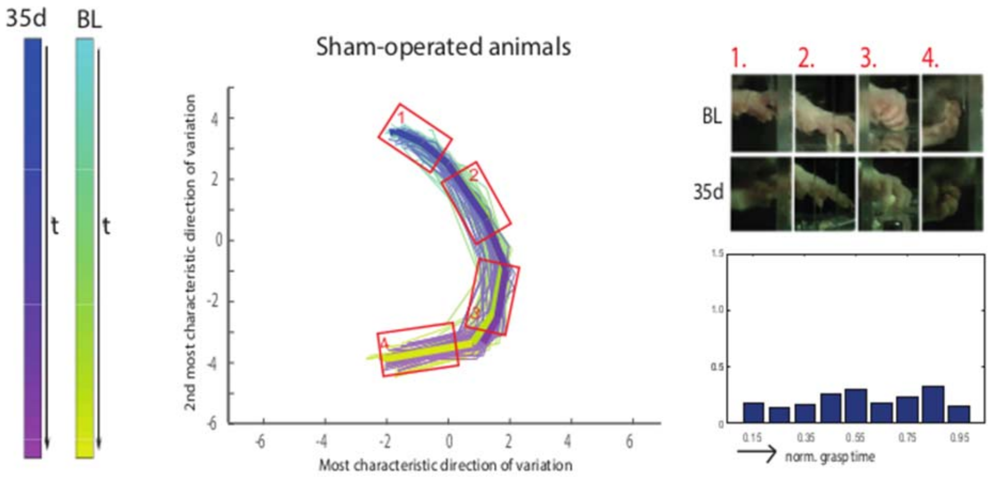


**Supplementary Figure 4.** Add-on for Fig. 3 depicting grasping trajectories of sham-operated animals at baseline (cyan-yellow trajectories) and 35 days after sham-surgery (blue-magenta trajectories). We found no posture differences between baseline and 35d after stroke for the sham-operated animals as shown in the trajectories (left panel), the bar plot (right panel), which summarizes how much behavior at baseline and at 35d after stroke differs at different stages of grasping, and selected frames of the grasping trajectories. BL=baseline.

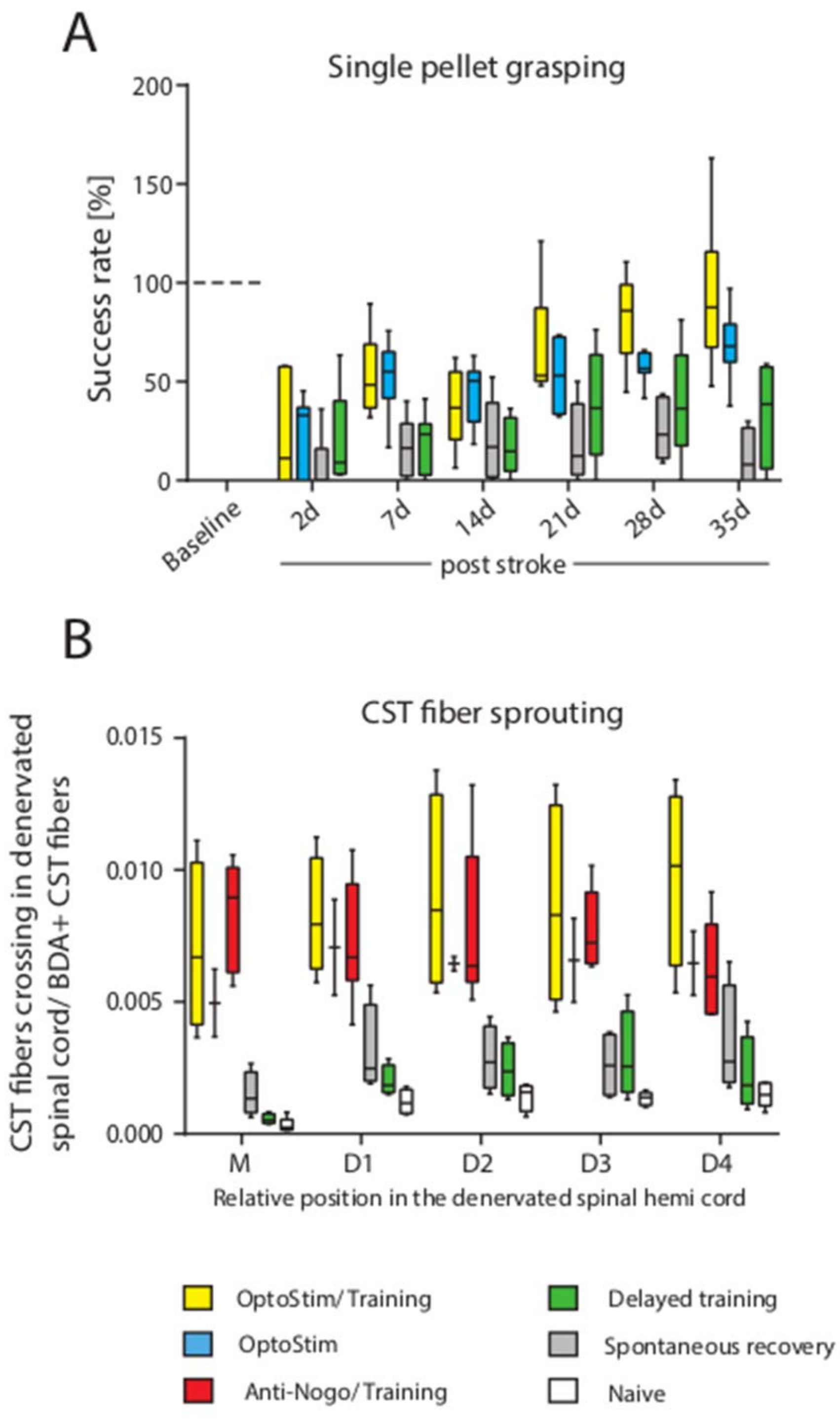


**Supplementary Figure 5.** (A) Box-whisker plots of Fig 1C depicting success rates in the single pellet grasping task relativeto baseline (100%; intact, trained) 2 days to 5 weeks after stroke. (B) Box-whisker plots of Fig. 4C showing fiber density of midline crossing CST fibers innervating the denervated cervical hemi cord, counted directly at midline (M), and at distances D1-D4 from midline.

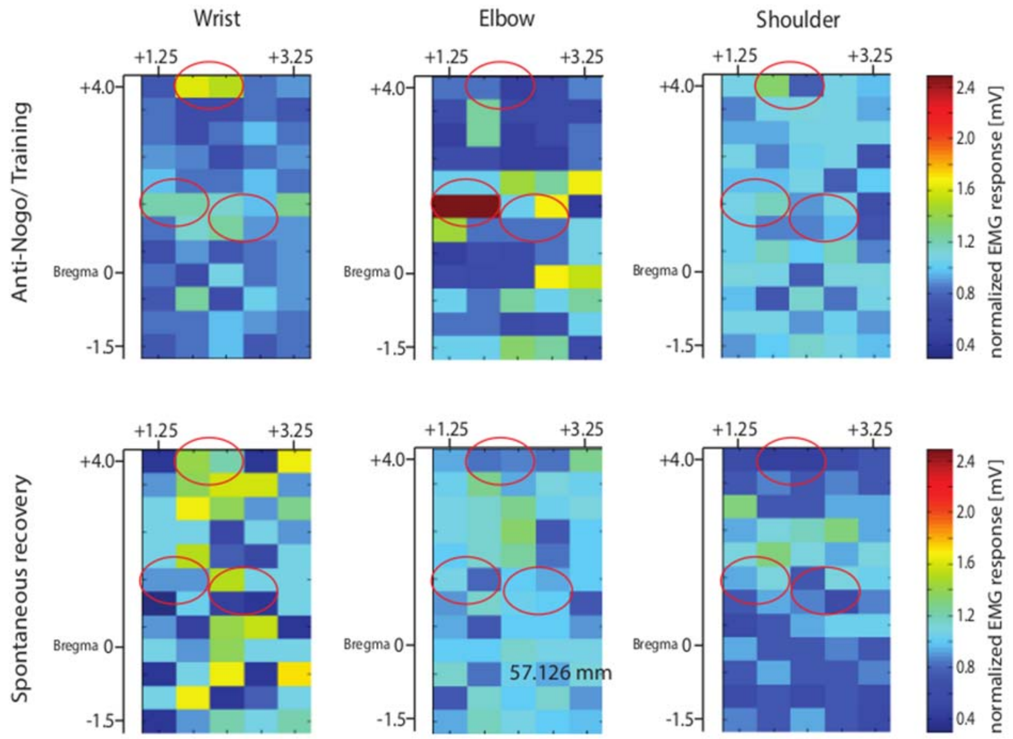


**Supplementary Figure 6.** Heatmaps of EMG responses in wrist, elbow and shoulder depending on the site of intracortical microstimulation using the following stimulation grid: 60 stimulation points, 80 µA, +4 to -1.5 mm AP and 1.25 to 3.25 mm ML relative to bregma. The intracortical microstimulation in particular revealed ‚hot spots‘ in the contralesional pre- and motor cortex for wrist and elbow responses in ‚Anti-Nogo/ Training’ animals (n=4). In contrast, in the ‘Spontaneous recovery’ group (n=3) only a diffuse cortical pattern for the evocation of EMG responses in the stroke impaired forelimb was found. The red circles indicate the previous positions of the three optical implants.

| | Position 1 | | | | Position 2 | | | | Position 3 | | | | all Positions | | | |
|---|---|---|---|---|---|---|---|---|---|---|---|---|---|---|---|---|
| | px | | mm | | px | | mm | | px | | mm | | px | | mm | |
| | mean | std | mean | std | mean | std | mean | std | mean | std | mean | std | mean | std | mean | std |
| **Sham-operated** | -0.768 | 1.3777 | -0.18 | 0.323 | 1.0421 | 2.0606 | 0.2442 | 0.483 | -1.218 | 1.75 | -0.285 | 0.4101 | 15.8573 | 3.944 | 3.7166 | 0.924 |
| **Anti-Nogo/Training** | 14.5792 | 4.2439 | 3.4166 | 0.995 | -8.47 | 3.1706 | -1.985 | 0.743 | 3.9768 | 2.543 | 0.9321 | 0.5961 | 15.6801 | 2.507 | 3.675 | 0.588 |
| **Spontaneous recovery** | -0.1308 | 0.7139 | -0.031 | 0.167 | 0.002 | 0.7004 | 0.0005 | 0.164 | 4.3346 | 3.014 | 1.0159 | -0.707 | -0.015 | 1.232 | -0.004 | 0.289 |

**Supplementary Table 1.** Quantified differences of the grasping length between light-on and light-off sessions supporting the visual results shown in Fig. 6. For every cohort and position a weighted mean/std x-coordinate of commonly occurring coordinates (local maxima of Fig. 6 B(I)/B(II), C(I)/C(II) and D(I)/D(II) after projecting the 2D matrix to the x-axis) during light-off and light-on sessions is calculated separately. After subtracting the outcomes of the light-off behavior from the light-on, the results are converted from pixel to mm. A positive mean value indicates longer grasps during a light-on session, while a negative mean value represents shorter grasps during light-on sessions than during light-off sessions. px=pixel, std= standard deviation.
