# Supplementary material for: Optogenetically stimulating intact rat corticospinal tract post-stroke restores motor control through regionalized functional circuit formation
Source: Nat Commun. 2017 Oct 30;8:1187. doi: 10.1038/s41467-017-01090-6 (PMC5662731; doi:10.1038/s41467-017-01090-6)
Supplement: Supplementary file 3 — Description of Additional Supplementary Files [file 41467_2017_1090_MOESM3_ESM.pdf]

Description of Additional Supplementary Files

File Name: Supplementary Movie 1

Description: Video showing a rat during optogenetic stimulation of the intact corticospinal tract with 10 Hz of 473nm wavelength LED light after a left hemispheric stroke impairing the right paw. Visible movements, partially with rhythmic jerking induced by the intact CST stimulation are seen, in particular in the healthy left paw.
